# Inevitable Decay: Debates over Climate, Food Security, and Plant Heredity in Nineteenth-Century Britain

**DOI:** 10.1007/s10739-018-9550-y

**Published:** 2018-12-11

**Authors:** John Lidwell-Durnin

**Affiliations:** History Faculty, University of Oxford, George St., Oxford, OX1 2RL UK

**Keywords:** Heredity, Thomas Andrew Knight, Malthus, Royal Horticultural Society, Botany, Grafting, Environmental history, Food security, Climate

## Abstract

Climate change and the failure of crops are significant but overlooked events in the history of heredity. Bad weather and dangerously low harvests provided momentum and urgency for answers to questions about how best to improve and acclimatize staple varieties. In the 1790s, a series of crop failures in Britain led to the popularization of and widespread debate over Thomas Andrew Knight’s suggestion that poor weather was in fact largely unconnected to the bad harvests. Rather, Knight argued, Britain’s older varieties—particularly its fruit trees—were coming to the natural end of their lifespans. At a period when Britain was trying to maximize its agricultural land usage, Knight campaigned that his fellow farmers ought to set aside land and resources in order to cultivate new varieties—an expensive and time-consuming procedure—in order to avoid disaster. In this paper, I argue that Knight’s lifelong commitment to his position demonstrates the role played by changes in climate and weather on popular understandings of plant heredity. Further, drawing upon the historiography of Britain’s climate and agriculture, I show that despite reliable weather and good harvests, Knight’s campaign survived for several decades before the continued health of Britain’s trees was finally treated as sufficient evidence to dispense with Knight’s warnings. This case provides a means of thinking about the history of heredity as it is shaped and impacted by changes in climate and local conditions.

## Introduction

The Golden Pippin is not, Mr. Lindley thinks, extinct, as some knowing persons pretend. (Anon., 1831, p. 159).

The year 1831 marked the 36th anniversary of Thomas Knight’s claim that all the golden pippin apple trees in Britain were soon to die. In those 36 years, farmers and botanists in Britain had remained largely divided over whether or not this popular variety was in obvious decline or in perfect health, despite the existence of numerous agricultural societies, magazines, and the introduction of rail networks. Communication in Britain’s agricultural networks improved, but the means of determining whether one of the most important apple varieties in Britain was soon to go extinct remained elusive. This controversy lasted for the rest of Knight’s life—it played out in the agricultural magazines, physiological and botanical treatises, in the pages of the *Royal Transactions,* and also in botanical illustrations. At issue was the sustainability of one of the basic principles of horticulture: propagation by grafts, a means of propagating varieties that was essential to the expansion of Britain’s fruit trees and also its growing arboretums (Elliott et al. 2011). Skill at grafting was respected in the eighteenth century, enjoying “the status of a liberal art as well as that of an empirical science” (Pacini 2010, p. 5). But the universal use of propagation by grafting meant that most of Britain’s cultivated trees were affected by new ideas about the longevity of grafts. For farmers like Thomas Bucknall, “They are doomed by nature to continue for a time, and then gradually decline, till at last the variety is totally lost, and soon forgotten unless recorded by tradition” (1796, p. 146). Britain was staring down the barrel of a massive die-off of its fruit trees—or so some believed.

How long, precisely, can a cultivated variety be relied upon in agriculture? In the final years of the eighteenth century, a series of harsh winters and wet autumns raised anxieties not only about climate and the best methods of cultivation, but also about cultivated plants themselves. Today, roughly 20 plant species count towards 90% of human calorie intake (Massawe et al. 2016, p. 365). Neglected varieties that have dwindled due to changes in agricultural practice are viewed today as invaluable to future food security. But similar anxieties plagued many at the close of the eighteenth century, when the question of Britain’s ability to feed itself was posed at the same time as farmers were beginning to debate whether certain key varieties had reached the natural end of their usefulness. Was it more important to rehabilitate older varieties, or to invest time and money in cultivating new varieties from seed? Despite widespread agreement in the agricultural press that bad weather and poor cultivation were the causes of bad harvests, some began to blame instead the varieties cultivated in Britain—not because they were unsuitable to the climate or soil, but because the varieties themselves were aging in the same way as does a person or animal.

The chief proponent of this idea was none other than the future president of the Royal Horticultural Society, Thomas Andrew Knight (1759–1838). Robert Olby views the early years of the Society as serving primarily the aristocratic interests of Britain’s landowners, believing that seed companies and nurseries only gained real inclusion in the functions of the Society after Knight’s tenure (2000, p. 1045). Such a view of the Society helps to explain how Knight was able to advance ideas that went against the grain of common practice for so long. Taking up the role in 1811, Knight worked for the rest of his life to communicate the importance of raising new, young varieties and asserting his own expertise on the question of good and bad practice in propagating trees. Fearful as many were of overpopulation, Knight warned that even the best methods of cultivation could do little to feed the expanding population if the varieties cultivated were not new. Older varieties, despite appearing healthy in places, were doomed to disease and decay—a view that, once expressed, found sympathy in Britain and in America (Pauly 2007, pp. 65–66). Twenty years after his death, Knight was dubbed by some “the champion of degeneracy” (Anon. 1857, p. 100). And if his warnings failed to drive farmers to abandon older varieties of cultivated plants, they certainly caused alarm, confusion, and debate on an international scale.

This article looks at an array of periodicals from the early nineteenth century to understand to what extent, if any, Knight’s warnings influenced agricultural practice. As I show, there is evidence within the wider agricultural press that there was increasing distrust and suspicion of the reliability of older varieties. People began to think of trees as having two ages: the age of the tree as an individual, but then in addition to this, the age of the tree as a variety. By drawing upon climate historiography, this article argues that this interest in the age of varieties was a direct response to poor weather and poor harvests. Despite the obvious challenges that the bad conditions of the 1790s posed to fruit farming, there was widespread willingness to think of agricultural deterioration as being a problem of varietal age, unrelated to climate. There was also a great degree of confusion and uncertainty. While efforts to compile statistics and agricultural reports were part of the wider French revolutionary wars effort, no comprehensive statistics were produced or available prior to 1866. Tithe records provide us with piecemeal indications about agricultural production for this era (Overton 1996, pp. 70, 74). However, these were difficult to examine for many of the people concerned about the health of Britain’s fruit trees, and many of those concerned seemed to have trusted the agricultural press to provide answers and guidance. As I show, it becomes clear from the sources that there was no accessible data on Britain’s fruit trees in terms of their health, their productivity, or the varieties that were most commonly planted. When Knight declared that the golden pippin trees were dying, neither the agricultural press nor the government had a real means of determining whether or not Britain’s grafted trees were healthy.

This confusion was key to the success of Knight’s ideas. Knight encouraged his readers to view new varieties—provided that they were developed utilizing his methods—as being naturally suited to Britain’s climate and categorically free of the diseases that he believed to be resultant from old age. Faced with an insurmountable problem (the threat of climate change), farmers seemed ready to adopt a view of their problems that promised that good or bad weather (an uncontrollable variable) wasn’t the deciding factor in a good harvest, but that the age of the varieties grown (a controllable variable) was of greater consequence.

## Climate

There were several notoriously bad years for farming in eighteenth-century Britain (and also in France). Historians looking at the Great Winter of 1709 have noted that most fruit trees in France were lost (including olive and chestnut trees), affecting food and income supplies for the poor for decades. From 1738–1742, autumn storms and cold winters led to the freezing of trees and the loss of crops in Britain, resulting in riots over grain exports (Michaelowa 2001, p. 207). Still, these disasters were regarded by many as natural processes. As Jan Golinski has shown, it was at the beginning of the eighteenth century that poor weather began to be regarded not as an omen or intervention from God, but as a natural event (2007, pp. 41–42). Golinski notes that British writers of the time viewed their climate as varied and challenging, and as “toughening the moral fiber of the inhabitants” (p. 58). As it challenged its human population, so too did this climate challenge imported varieties of fruit. Varieties that were deemed to have been adapted successfully to Britain’s climate and soil were celebrated and traded commercially. Efforts to document and share methods of acclimatizing foreign plants date back at least to Pierre Belon’s garden of acclimatization, established in the 1500s (Elliott 2009, p. 23). Centuries later, there was a pressing need to acclimatize European plants to Australia’s climate and soil (Finney 1984). Still, prior to improvements in glass manufacturing in 1847, glass houses remained expensive and difficult to construct, with irregularities in the glass causing leaves to burn (Elliott 1986, p. 17). Investment and labor were involved in most efforts to acclimatize foreign varieties, leaving such experiments to a wealthy few.^1^

Hubert Lamb suggested that societies have usually taken improved climate for granted, only noticing and recording the effects of poor climate (1995, p. 1). In strong support of this thesis, in the 1790s a series of bad harvests and poor weather led the British government to conduct a series of surveys on its agricultural production. The ability of Britain’s agricultural produce to support its own population was cast into doubt. In such an intellectual climate, areas that experienced poor yields or disease gained closer scrutiny. As Minchinton noted (1953, p. 29), 1795 was particularly wet and cold; it was a year that marked considerable disease among the fruit trees belonging to Thomas Andrew Knight and his neighbors in Herefordshire. The 1790s themselves were particularly disruptive and witnessed price-fixing riots across Britain. As Overton argues, the markets were in flux during this period, with small consumers bearing the brunt of the changes (1996, pp. 189–190). The conditions of 1795 were so poor that the newly-established Board of Agriculture took steps to arrange for the first systematic efforts to calculate the self-sustainability of British agriculture (Minchinton 1953, p. 31). With Europe at war, the fear of food shortages in Britain was pronounced, and Joseph Banks’s interest in Knight’s ideas was certainly part of this larger question as to what dangers and challenges stood in the way of a self-sufficient Britain. In this period of crisis, Knight’s expertise fit into Banks's wider concern to promote agricultural self-sufficiency (Bord 2009, p. 27–28).

When such efforts yielded less than the desired certainty, Britain took the step of banning the export of grain (Minchinton 1953, p. 35). The continuing rise in population had, since the 1500s, affected prices for some time. As Behringer explains, “The population grew faster than the production of cereals. Agriculture reached its limits in the late sixteenth century … and the poor could no longer feed themselves at affordable prices” (2010, p. 109). While many factors impacted these price fluctuations, Behringer shows that the price of grain more than doubled around the turn of the century, before quickly declining in price (p. 110). The failure of crops due to climate had an immediate effect upon parishes administering allowances to the poor, the allowances being “a necessary response to the sharply worsened circumstances after 1790” (Williams 2004, p. 82). Knight’s contentious ideas about grafting and the decay of older varieties were put forward during a period when the climate’s effects on agriculture were threatening economic and social crises, and they contradicted the popular belief that the bad weather and poor harvests were connected.

Much of the historiographical interest in the climate and agricultural production of the 1790s relates to the publication of Robert Malthus’s *An Essay on the Principle of Population* (1798). As Hurzel notes, poverty was rapidly increasing in the years building up to Malthus’s publication, and his *Essay* ran against the predominant mercantilist ideas of the benefits of a growing population (2006, p. 2). Yet Malthus was never critical of the ability of Britain’s farmers to employ the most rational methods for farming; on the contrary, he had great faith in British agriculture, a faith shared by many during his lifetime (Hilton 1977, p. 23; Salvadori and Signorino 2015, p. 160). A culture emerged that was highly skeptical of the sustainability of old practices and models and, at the same time, positive and hopeful that innovation would meet all the mounting pressures on the country. Poverty and hunger were inevitable consequences of current practices; but this was a natural danger, and one that could be postponed indefinitely with the aid of science. According to Maureen McNeil, in this way both Malthus and the physician Erasmus Darwin “naturalised the poverty and suffering in Britain” (1987, p. 40). Knight also was deeply committed to this way of thinking. Towards the end of his life, in 1834 Knight continued to advance Malthusian attitudes, explaining that, in his opinion, “It is obvious enough that, at present, the British islands do not support the present population, whilst much of it remains unemployed, or not fully employed” (p. 42). He had taken a keen interest in the application of the Poor Law in Herefordshire and hoped that by setting the poor to work, Britain might finally reach self-sufficiency. While Knight and Malthus did not correspond, they shared very similar attitudes towards the immediate problems facing the country and only disagreed on what kind of experience placed a gentleman in a position of authority on the topic.

In the reports published by the Board of Agriculture, some observers shared Knight’s opinions on the dour future of key varieties. Knight found his ideas fully supported in the account of Herefordshire’s agricultural development of 1813, where the author explained to the Agricultural Board that the idea of developing new varieties had found widespread success. “This experiment has been adopted on a large scale by several planters, has hirtherto promised the fullest success, and has further the sanction of that period, in which orcharding received particular attention” (Duncumb 1813, p. 81). Kent also reported the influence of Knight’s ideas. “It is to be observed, that, in Kent, as in the cider countries, the old favorite kinds are gone, or going off. The Kentish pippin, and the golden rennet, are no longer in propagation, and the golden pippin is become unproductive” (Marshall 1798, p. 305). The views were shared by others. Even Knight’s enemies admitted that something was causing widespread deterioration in Britain’s fruit trees. The agricultural publisher James Anderson, set to become one of Knight’s greatest critics, explained: “The golden pippin is justly esteemed a very valuable fruit but the tree which produces it is weakly, and subject to canker. The fruit itself loses its sharpness in a few months, and then becomes insipid” (1802, p. 17). Anderson, like Knight, called for experimentation with raising new varieties from seed. But whether one blamed climate or something internal to the variety, there was widespread agreement that something was going badly wrong with Britain’s fruit trees. Decades later, the poor conditions were still remembered in agricultural articles that discussed Knight’s ideas:

The circumstance which suggested the idea to the worthy president was not only apparent in Herefordshire, but, at that time, all over the kingdom. The old golden pippin, the styre, and fox-whelp, were failing in all directions around Mr. Knight’s residence, in Herefordshire, and adjoining cider countries; and the first of these, a great favourite everywhere, became almost barren, and was visibly failing all over the kingdom. (Anon. 1837, p. 355)

To Knight and to many others, it seemed as though the golden pippin had reached its natural end, and the fears continued into the nineteenth century. As one observer commented, “All the favourite apples of the last century are gradually deteriorating. The Golden Pippin has not a fourth of the size described by the old writers on gardening; and our hopes for new and excellent varieties must rest upon enlightened experiments on seedlings” (Anon. 1807, pp. 51–52). Further complicating the controversy were widespread confusions over identifying varieties. As Sarah Dewis has argued, the plant trade in Britain and Europe opened opportunities for the misidentification of plants and trees, exacerbating problems within the classification system (2014, p. 119). While Knight always presumed that his readers were confident in the identities of their trees, in reality it could prove exceedingly difficult to identify the variety of a given tree with real certainty. Widespread calls for more rigor and scientific practice (whatever that might entail) were common in the agricultural and gardening press (Lidwell-Durnin 2018). The proliferation of agricultural and farming periodicals during this period created numerous platforms from which the causes of decay and deterioration were debated, but it was because of the ambition of Joseph Banks that such authority could be concentrated in one official body: the Royal Horticultural Society.

Knight’s position on the necessity of widespread reform in cultivation promised higher yields of fruit, and he threatened continued declines in yields unless his positions were recognized and his methods adopted. In doing so, Knight’s position fit within the behaviors recognized by historians of climate: “Under the pressure of population and in the face of a declining per capita availability of energy and rising prices, any traditional agricultural society reacts by trying to intensify the use of land” (Kander et al. 2015, p. 103). Knight’s warning that failure to act on his recommendations came at a key moment where rising prices and poor weather had induced widespread anxiety. The security of the near future mattered to all, and did for many decades. As one proponent of Knight’s ideas explained, “I believe all will agree, that in adopting his theory, we adopt the safest course” (Kenrick 1833, p. xvii).

## Degeneration

How, precisely, did Knight come to “champion” degeneracy in the 1790s? Why did he propose an understanding of the poor harvests that entirely removed the effects of climate from consideration? It was an unusually hard position for an era when most of the views on heredity, variation, and degeneration were deeply committed to the effects of climate. Knight’s hard views on heredity and degeneration also pre-date the height of such concerns in the latter half of the nineteenth century. For Daniel Pick, the events of 1848 established a deepset anxiety over degeneration, leading to a “hardening” in medical views on hereditary illnesses (1989). Staffan Müller-Wille and Hans-Jörg Rheinberger have recently argued that anxieties and questions concerning heredity have always operated in the background, operating the same influence on society that can be attributed to religious and economic beliefs (2012). As they have shown, eighteenth-century botanists regarded climate as having a continuous effect on plants and used degeneration as a means of explaining the inherited effects of climate on a variety of species (2012, p. 61). Of course, such use was not limited to the plant realm. Georges-Louis Leclerc, Comte de Buffon, Johann Friedrich Blumenbach, and Immanuel Kant all invoked the degenerating effects of climate in order to explain the variety of human races (Gould 2006, p. 71; Mensch 2013, p. 101; Richards 2000, p. 20). Knight’s own anxieties are situated in a complex era. They belong to wider fears of food security during the War of the First Coalition, but they also put Knight at odds with many of the main tenets of degeneration theory—most importantly, the idea that degeneration and deterioration stem from climate and cultivation. Knight was, of course, deeply concerned about cultivation and its importance to feeding Britain, but here, in his most infamous idea, he argued against the usefulness of cultivation or acclimatization entirely.

How did Knight’s ideas fit popular understandings of heredity in this period? Desirable traits in fruit like sweetness, color, and texture were easily lost if trees were propagated by seed, but preserved when propagated by grafting. In the early decades of the nineteenth century, many nurserymen and gardeners presumed that characteristics of the root stock and the graft were capable of being transmitted, producing what were referred to as “graft hybrids” (Lidwell-Durnin 2018). By means of grafting, it was hoped that humans could produce lasting changes for the better in cultivated varieties. During this era, as Marc Ratcliff put it, gardeners and cultivators “understood their practices of transforming sorts and species [as] the influence of human art on nature” (2007, p. 229). Knight shared in the belief that human influence could improve cultivated varieties, but by declaring cultivated varieties as mortal he put great limits on what cultivators could achieve. Within medicine, as Waller (2002) has shown, an enthusiasm for understanding incurable illnesses as hereditary provided medicine with a much-needed framework for communicating its limitations. Knight was convinced that plant breeding and animal breeding followed the same laws. “There is so much in common in animal and vegetable nature,” he wrote to Banks in 1797 (T.A. Knight to J. Banks, Jan. 27th 1797, p. 3).^2^ He explained that he was “fully aware of the danger of error in transferring the phenomena of one class of organic beings with another,” yet clearly he viewed his animal and plant breeding experiments as contributing equally to an understanding of one natural law (MSS/KNI/Jan. 27th 1797, p. 2). Hereditary illnesses, after all, are written into the constitution of the individual. The best way to steer towards a world without illness for both the plant and animal realms was proper management and proper breeding. Darwin disagreed with Knight’s views on the individuation of trees, but he certainly was willing to regard canker as an hereditary illness—such an approach still agreed with Knight’s observations and some of his recommendations (Darwin 1800, p. 324).

Knight felt that his experiments and observations constituted a real emergency: the older varieties doomed to die, he reminded his readers, “formed the largest orchards in this country” (1795, p. 290). These trees were in fact a single individual, all the same age and all doomed to die when the natural lifespan of an apple tree was reached, no matter how “old” the individual trees might be considered to be by the farmers that had planted them. And while Knight took advantage of the poor conditions of 1795 to put forward his ideas, he had several years’ worth of experiments practiced on his own farm to support his ideas (Kingsbury 2009, p. 80). He explained that an easy way to test his ideas about grafting was to take cuttings from very young trees grown from seed—too young to bear fruit—and to graft these onto mature stock. Repeating this experiment several times, Knight claimed that none of these grafts produced fruit, further evidence in favor of his views (1795, p. 292). The canker Knight observed affecting his trees—no matter the age of the graft—was the other important base of evidence. “Canker” was already a term already associated with old age. As Knight developed his ideas, he sought to unify his work on plants with the human case: “Is it very improbable,” he posed in a later article, “that the natural debility of old age of trees and of animals, may originate from a similar source?” (1810, p. 183). Such views drove against the popular belief that trees were potentially immortal. “Before Mr. Knight advanced his theory,” one farmer would later comment, “it was the general belief that when a good variety of fruit was once originated from seed it might be continued by grafting and budding for ever” (Saul 1857, p. 364). In 1662, John Evelyn reported trees reaching an age of 2500 years (1662; 2013, p. 190). Augustin de Candolle speculated that some trees might be capable of living forever, if they escaped all accident and disease (1832, p. 1003). Knight gave apple trees a century or two at best and was skeptical of longevity in any plant form—even varieties propagated by seed. Contemporaries were surprised at the short life spans that Knight predicted for trees. John Lindley, who began his career as an assistant to Joseph Banks, commented later that “I cannot for a moment agree to such an opinion,” in reference to Knight’s enduring ideas on the subject (1831, p. 16).

Knight also worked his beliefs into his vegetable physiology. As he developed his ideas on the circulation of sap, Knight used this research to support his earlier claims about the lifespan of grafted trees. In his papers on the subject of sap, Knight reminded his readers that the illnesses affecting the golden pippin aren’t observed in the stock. He reasoned that an over-abundance of nutrition provided by the young root system overwhelmed the aged constitution of the graft, resulting in many of the illnesses perceived in older varieties like the golden pippin (1810). While there was resistance from authorities like John Lindley, Knight built his wider views on plant physiology around the insights he had obtained from the lifespan of grafts.

The physiology of trees, an important subject in the early decades of the nineteenth century, wasn’t without immediate and important consequences for society. Fears of “timber famine” had been integral to the rise in coal use in the seventeenth century (Kander et al. 2015, p. 103), and shortages of good timber remained a key topic in Britain well into the 1830s, prompting Patrick Matthew to publish on the subject of arboriculture and the importance of timber for the navy (1831). Timber imports from the empire helped to meet the ever-increasing demand for soft timber, but many voices in Britain complained that fondness for “overripe” mature hardwood trees was dragging Britain behind the manufacturing and output of other European countries (Winter 1999, p. 90). Anxieties over timber led the government to invest £1500 into William Forsyth’s “plaister,” a treatment that he claimed could be applied to old trees and cause new growth where decay had set in (Simmonds 1941; Meynell 1979; Mylechreest 1984; Jacques 1996). As Forsyth claimed to his readers, the royal forests belonging to the crown were diseased and in poor condition, and commissioners appointed by Parliament sought a means of saving them from disease. Forsyth claimed that up to one-third of the harvested timber was, in fact, damaged ((1791), 1818, pp. 412, 413). His plaister was to be a panacea. For Knight, of course, the idea that a plaister could treat the illnesses of old age and restore trees to their youth was ludicrous, as I will discuss below. Forsyth found many adherents and proponents of his methods, and the popularized recipe, consisting of clay, slaked lime, wet ashes, and fresh cow dung was used for many decades in the hopes of repairing the health of trees. However, growing skepticism of his claims and methods no doubt contributed to the rise in popularity of Knight’s more pragmatic views. As Forsyth’s reputation dwindled, Knight was able to position himself as the foremost institutional authority on the health and cultivation of Britain’s trees. Anxieties over the health of Britain’s forests and its ability to feed itself would only increase as the Napoleonic wars continued. By championing the cultivation of new varieties (and by distancing himself from the aura of quackery that surrounded Forsyth), Knight introduced new fears but also proposed a means of assuaging them that was built upon familiar and traditional practices.

## Reception

Knight may have been largely unknown to the agricultural world outside of Herefordshire when he published his first treatise, but for those who disagreed with his views he was easily portrayed as a wealthy amateur, someone lacking in the kind of practical experience that was valued in the agricultural press. His attacks on William Forsyth only served to further the public opinion that Knight was not only in error, but also untrustworthy. The agricultural writer James Anderson complained that “fondness for this hobby has biased the judgment of this respectable gentleman” (1802, p. 535). Knight was infuriated and complained to Banks: “in a letter I have been compelled to address to Dr Anderson in answer to accusations made against me by him” (MSS/KNI/June 13th 1802, p. 33). Erasmus Darwin, seeing his own views on the relationship of the bud to the parent tree falling under attack, explained to his readers that: “This degeneracy of trees or perennial herbaceous plants propagated by buds or root-scions is not I think to be ascribed simply to the age of the original seedling-tree, because each successive generation of buds or bulbs are as distinct from the parent, as the generation by seeds” (1800, p. 96). The disdain was mutual. “I have read some time Dr. Darwin’s *Phytologia*, as such talk of his *Zoonomia* as relates to vegetation,” Knight wrote to Banks. “All his works appear to me to convey much important information mixed with a very large part of ingenious nonsense” (MSS/KNI/July 17th 1800, pp. 18–20).

The controversy with Forsyth put Knight and his claims about the age of trees in the plain light of other observers. Anderson, a longtime proponent of Forsyth, took it upon himself to visit Knight and proclaimed that his golden pippin trees were in perfect health (1802, p. 527). If Forsyth could heal his trees by the application of his plaister, then surely much of what Knight believed was wrong, for the plaister could hardly be said to be making old trees young. For Mylechreest (1984) and Simmonds (1941), this disagreement was central to the early shape of the Royal Horticultural Society. But it also introduced a significant conflict within the realm of agricultural advice. On one side, trees propagated by grafting could be expected to lead long lives. On the other, whole varieties were slated for imminent deterioration with no hope of intervention. Until Forsyth’s death in 1804 and Anderson’s resignation from the Society, Knight felt unable to act on Banks’s encouragement that he become involved (Mylechreest 1984, p. 135). But once he was brought into the Society in 1805, his views on the deterioration of varieties quickly overtook the influence of posthumous copies of Forsyth’s own ideas.

If Knight had failed to convince Darwin and many of the leading agricultural writers, he had clearly persuaded Banks. The controversy with Forsyth had stemmed entirely from Knight’s insistence that one could not treat the symptoms of old age, and that any claims to have succeeded in doing so were false. Nonetheless, Banks was able to secure the presidency of the Royal Horticultural Society for Knight, lending a new and significant authority to his claims about the nature and lifespan of trees. With the appearance of the first issue of the *Transactions of the Horticultural Society of London* in 1807, the impending doom of Britain’s oldest and most widely-cultivated varieties was given new authority. “For I am thoroughly convinced,” Knight reminded everyone in the first issue of his new publication, “that the graft derives nutriment only, and not youth, from the young stock in which it is inserted; and that with the life of the parent stock the graft retains its habit and its constitution” (1807, p. 62). As it remained impossible to reach consensus on the actual condition of the golden pippin across the country, Knight’s views continued to gain followers and adherents, as he waged his campaign against the older varieties cultivated in Britain. Arboriculture remained largely a topic of concern only to wealthy landowners, as it was not until the 1820s that inexpensive gardening magazines began to promote the science at a more accessible level (Dewis 2014, p. 65). Still, Knight’s letters and ideas were frequently repeated in the cheaper magazines and periodicals of the time. Dictionaries and encyclopedias of gardening also began to include Knight’s views on how trees produced by grafting would age (McDonald 1807, p. 360).

As James Winter has shown, one of the most important aspects of the history of agriculture in nineteenth-century Britain lies in the fact that “so few owned so much of the land surface” (1999, p. 4). The financial interests of these landowners were balanced, Winter notes, with paternalistic attitudes towards the estate, maintaining much of the character of the older countryside in the interests of game hunting and rural life. Knight’s vision of experimentation and investment in the cultivation of new varieties presented a conflicted future to these powers. It preserved the characters of cider country and resisted shifting towards more financially viable uses of land, certainly, but obvious expenses would be entailed if Britain’s landowners were to dedicate land and resources to growing trees from seed in search of new varieties. At the same time, could landowners afford the costs of nurturing aged varieties? As Thomas Bucknall complained:

It is a known fact, and mentioned in the seventeenth volume of the Transactions, that after the debility of age has actually taken portion of any variety, it will yet thrive by being placed against a southern wall, and treated as wall-fruit. Who, however, can afford to raise cider at that expense, except as matter of curiosity? (1796, p. 162)

Once Knight’s way of viewing the lifespan of trees gained popularity, there seemed to be rising costs for production no matter whether one tried to maintain the productivity of existing trees or if one tried instead to contribute to the development of new varieties.

Knight’s labors to discover new varieties from seed had, by 1815, become widely known. As the gardening and agricultural press developed, Knight’s ideas and positions gained a new sphere for debate. These popular titles were often tied to the business of seed distributors and nurseries who advertised in their pages, and so debate and interest in new varieties were central themes. Eleanor Anne Porden, writing in the same genre of scientific poetry as Erasmus Darwin, learned of Knight’s ideas through the lectures of Humphry Davy and the botanist James Edward-Smith (Smith 1815):

How, grafted on its stock, the crab will bear

The sweeter apple or the juicy pear,

But gradual as the parent grafts decay,

The sympathetic offspring fades away.

(Porden 1815, p. 29)

Porden championed Knight as a hero. Support and diffusion of the idea that the old varieties were dying and that new varieties needed to be swiftly developed were common in the anonymous articles that filled the pages of these publications. His efforts were often explained to readers as being heroic (if not extreme):

…His chief object was to obtain improved variations of the apple, to supply the place of those which were diseased or unproductive, by being cultivated beyond the period apparently assigned by nature. (Anon. 1800, p. 245)

It was also around the time of Porden’s publication that Knight’s new varieties were gaining popularity. Moreover, Knight’s suggestion that old varieties were not to be trusted was having an effect the popularity of older varieties. In *The Caledonian Horticultural Society*, a member commented in reference to Knight that “now nothing is heard of but new kinds to be obtained from seed, as the only means of procuring trees healthy, and free from canker” (Sang 1812, p. 337). But it wasn’t the novelty of these varieties in and of themselves that led to their discussion. Rather, the threat posed by declining varieties was always placed at the forefront:

…few experiments have been made to obtain new productions of this sort, though some, especially of the apple tribe, are evidently declining; and, since almost every ameliorated variety of fruit appears to have been the result of accident, we may fairly infer with Mr. K. that an ample field for discovery lies open.” (Anon., 1809, p. 267)

Another factor might explain the continued interest in Knight’s ideas. As Lamb (1982, p. 229) has shown, the years 1810–1819 saw the coldest winters in Britain since the 1690s. Most likely these winters were caused by the eruption of Tambora in 1815 and an unknown eruption of similar scope in 1809 (Cole-Dai et al. 2009, p. 16). England saw an overall rise in agricultural productivity during this decade (Tilly 2005, p. 121), but cold winters still posed challenges to fruit trees, with canker being encouraged by colder, damp springs.

**Fig. 1:**
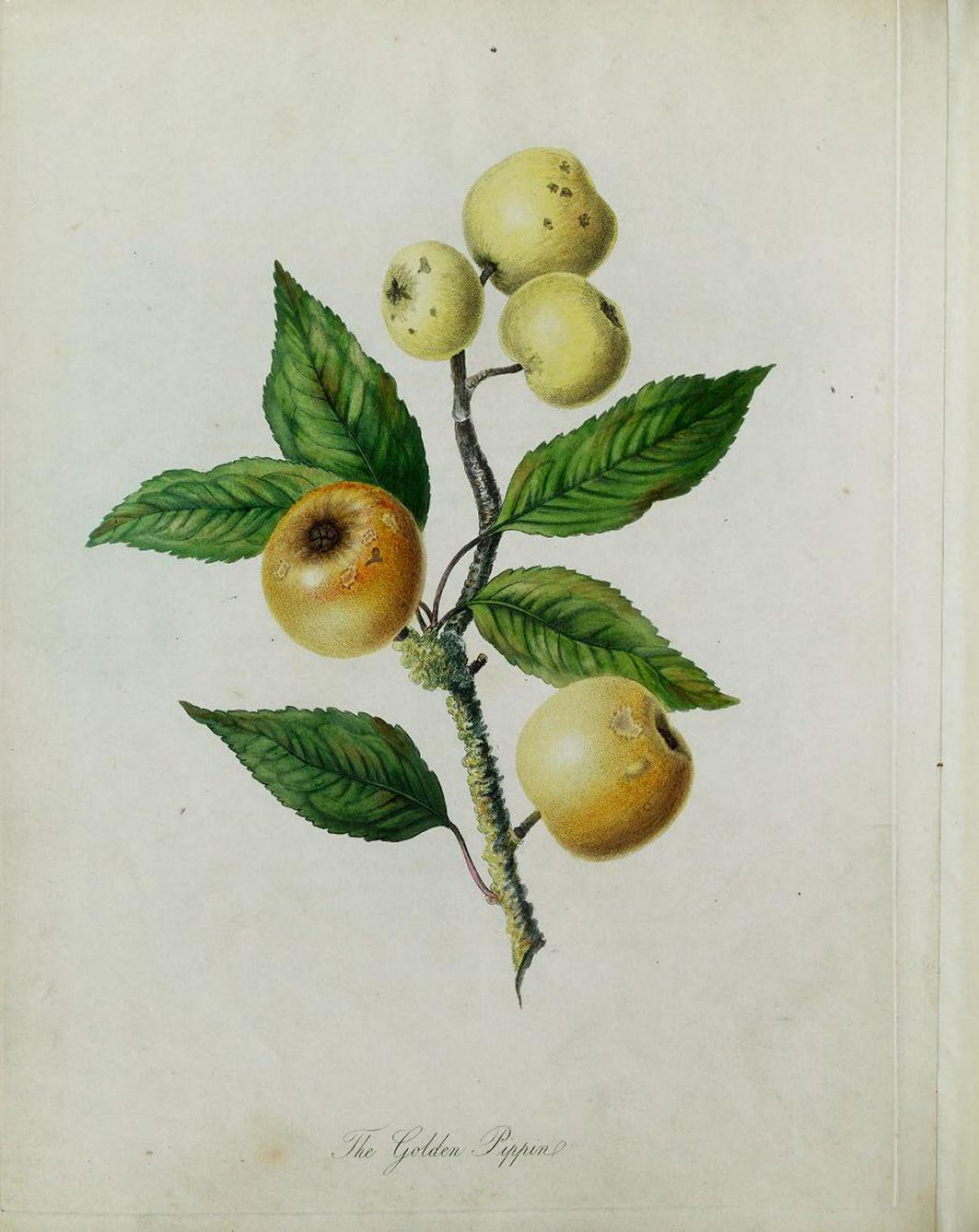
Illustration of the Golden Pippin, in *Pomona Herefordiensis* (1811). Note the inclusion of blemishes, mold on the leaves, and lichen on the branch. Image taken from the Natural History Museum Library, London. See: Knight (1811)

**Fig. 2:**
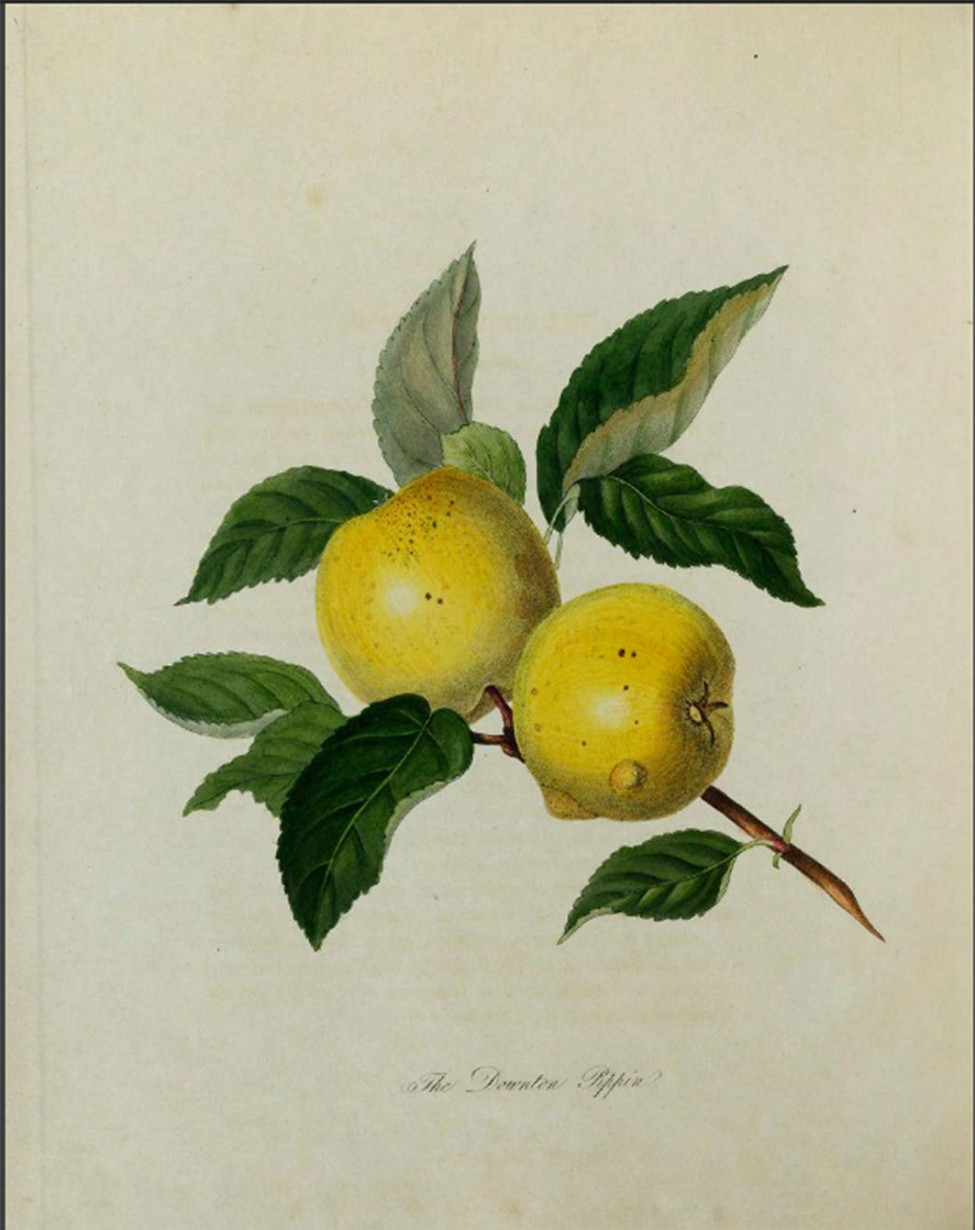
Illustration of the Downton Pippin, in *Pomona Herefordiensis* (1811). As contrasted with Fig. 1, the illustration of the Downton Pippin shows the tree largely unblemished and free from lichen. Image taken from the Natural History Museum Library, London. See: Knight (1811)

In the year 1811, Knight became president of the Horticultural Society. But he also used this occasion to publish an illustrated guide to the trees of Herefordshire, titled *Pomona Herefordiensis.* Since 1795, debate had continued in the agricultural publications of Britain as to whether or not the golden pippin and other varieties were in a state of decline, with no agreement as to whether or not the trees looked healthy or debilitated. It seemed (unsurprisingly) that there were plenty of deteriorating trees, but also many healthy ones to be found throughout the country. In *Pomona Herefordiensis,* Knight attempted to classify and demonstrate the visual differences between older varieties and new varieties recently developed from seed. As Fig. 1 shows, Knight depicted the golden pippin covered in lichen (a sign of old age), with signs of mildew on the leaves and scabs on the apples. As Knight explained in the accompany text, “Owing to the debilitated state of the variety, in which the vital principle appears to be nearly expended, much of the fruit generally remains imperfect and immature, as represented in the plate” (Knight 1811, p. ii). For the new varieties being grown in Herefordshire, no such blemishes or imperfections were added. These were depicted as free of disease. “The decay of every variety of the apple and pear,” Knight explained to his readers, “which has long been cultivated, is now very generally admitted.” More significantly, Knight had provided a guide to identifying these differences to readers, “particularly if he be no botanist” (1811, p. ii). Contrasting the golden pippin with an illustration of Knight’s own variety, the Downton pippin (Fig. 2), shows the degree to which the health and vigor of the younger variety was presented. The leaves are unblemished, and aside from a few imperfections in the fruit, the plant is healthy and vibrant. “The drawings, however, are perfectly accurate,” Knight explained to assuage any scepticism, “and are portraits of branches selected by myself from young trees in my nursery” (1811, p. ix). When William Hooker (the illustrator) published his *Pomona Londinensis* in 1818, he omitted the golden pippin from consideration, including instead new varieties. When discussing another variety (the kerry pippin) that Knight also believed to be near death, Hooker explained that “I am informed by Mr. Knight, that trees in his possession, which are older, are much infested with canker” (Hooker 1818, p. xx).

Through these illustrations, Knight was able to diffuse a canonical guide to the signs of old age, and he was able at last to provide visual evidence of how Britain’s older varieties were dying. As Anne Secord has argued, this kind of instructional illustration, aimed at readers who had direct access to the plant being depicted, was common in the early nineteenth century (2002, p. 32). Knight depended for legitimacy upon practitioners and followers of his thinking and methods, and images provided Knight—as they did botanists—with an opportunity to train others in how to see and observe the key features of aging trees that he hoped to communicate. With public libraries still uncommon, few readers would have had access to illustrated botanical studies. However, things were fast changing: the teaching of botany increased after the Apothecaries Act of 1815, and Knight’s own work appeared just as the public and academic demand for botanical illustrations began to widen (Secord 2002, pp. 41, 42). As Iain Watts and Jonathan Topham have independently argued, the early 1800s were a period in which reprinting was particularly important to the emerging scientific journals of the day (Watts 2014, pp. 398–399; Topham 2013). Readers who didn’t have access to Knight’s treatises or to his publications in the *Transactions* still regularly encountered reprinted material from these works within the cheaper scientific journals and magazines.

Where Secord has discussed the importance of such images in helping the wider public to engage in identification and classification, here the images serve a different purpose. Knight’s illustrations are intended to serve as a guide towards identifying a *dying variety*, and not just illness in one particular tree. They provided a visual guide to distinguishing youthful and healthy varieties from older ones, largely irrespective of the age of the tree.

Knight also gained popularity with a new generation of scientific writers. Humphry Davy, who particularly enjoyed fishing on Knight’s estates, became a lifelong proponent of Knight’s views on the nature of plant life and popularized them in his lectures. Eager to please Knight, Davy wrote to him in 1808, saying, “You have created almost all the science we have on the (proofs upon that interesting) Subject” (H. Davy to T.A. Knight, 1808–11–03).^3^ What drew Davy (beyond an appreciation of Knight’s grounds) to champion his ideas? Davy, like many of his era, mourned the passage of time and viewed inevitable decay as part of the natural order (Golinski 2016, p. 166). As Davy explained in his lectures, “The decay of the best varieties of fruit-bearing trees which have been distributed through the country by grafts, is a circumstance of great importance. There is no mode of preserving them; and no resource, except that of raising new varieties by seeds” (1813, p. 228; 1817, p. 461). Davy’s popular writings brought Knight’s position yet another means of authority, and for a time the idea that older varieties degenerated as a matter of course became a common theme in agricultural magazines. “It is well known,” an anonymous contributor explained in an article on the causes of disease in trees, “that some fruits are no longer what they were: that the progeny of others appear to have greatly degenerated” (Anon. 1815, p. 640).

The theory lately published as to the period of existence of fruit and other trees, for which the world is indebted to Mr. Knight, is fixed on reasonable and true principles, and we see it more and more evident as time elapses; we also observe a gradual change in many of our fruit trees for the worse. [….] but notwithstanding, I observe such kinds in other places healthy and thriving, so that I am of opinion, that these varieties are capable of being brought round again, equal to what they have been, and what they were with us thirty years past. I would therefore wish to caution the growers of fruit from falling into the extreme. (Salisbury 1816, pp. 99–100)

Other trees began to be viewed through the lens of Knight’s ideas. They provided a clear means of thinking about illness, particularly of illnesses that didn’t seem to provide other explanations:

On regretting the appearance of some stately chestnut trees near Mr Doveton’s country house, which seemed to have been sometime dead, I was assured, that within these last four or five years, the greater number of the chesnut, of every age in this Island, have either died or are dying. On inspecting several at the Plantation House, in both these states, I could perceive no insects, nor any other source of destruction, beyond the ordinary indications of decay in an aged tree. (Anon. 1815, p. 640)

By 1820, a quarter-century without disaster had elapsed since the first warning. Humphry Davy continued to support Knight’s ideas and to include them in his lectures, but other authorities were beginning to challenge Knight based on the fact that the doomed varieties were still living. It is difficult to directly assess to what degree farmers were adopting Knight’s recommendations, but there was evidently enough effect to the fruit market to prompt the botanist Henry Philips to include what he called “a digression” in his *Pomarium Britannicum,* containing an extended and detailed defense of older varieties and propagation by grafting (1820, p. 47). Arguably, Philips’s *Pomarium Britannicum* was never destined to reach many of the readers that might have a stake in concerns about the vitality of Britain’s fruit trees, but his discussion of Knight’s ideas was frequently excerpted and repeated in the agricultural press. For Philips, the sight in Covent Garden market of “a great quantity of the real Golden Pippin in a perfect state” provided the key motivation in questioning Knight’s ideas (p. 46). Through his networks, Philips determined that most farmers agreed there had been an outbreak of canker and bad years, but that by 1818, things had greatly improved. As Philips argued, he hoped to “prevent if possible our best apples from being stigmatised as a decaying fruit and unprofitable to the grafter, which would be the cause of their becoming scarce, and in time, totally lost” (pp. 47–48). Here came a new concern: that the president of the Horticultural Society was directly responsible for encouraging farmers to abandon older varieties that now ran the risk of dying out—not from old age, as Knight himself would have it, but from neglect. Philips promoted Knight’s new varieties in his book and included illustrations for his readers to better aid them in experimenting in breeding new varieties, but his goal in doing so was to increase the number of varieties cultivated in Britain, not to dissuade farmers from cultivating older varieties.

Knight himself never altered or revised his opinion, even when acknowledging that changes in the climate bore some impact on trees. In 1812, when discussing varieties, Knight helped himself by describing fruit varieties cultivated in Britain as having ages. “The Elton Pear,” for Knight, was a tree that “will probably remain in health for three centuries” (p. 2). He remained apparently unmoved by the increasing doubt about his claims; it remained central to his descriptions of trees and to his instructions for British farmers. Knight was also attentive to Britain’s shifts in climate. In a paper read in 1829, Knight remarked, “that our winters have grown warmer, it cannot, I think, reasonably be rejected” (1841, p. 307). He observed that trees that had been cultivated to suit Britain’s climate 50 years ago would struggle, and that new varieties ought be cultivated to take their place, but he never allowed that these shifts in climate might be related to the difficulties observed in the varieties he doomed to old age. In 1831, Knight read a paper at the Horticultural Society on the subject of “Prolonging the Duration of Valuable Varieties of Fruit,” where he reiterated his belief that there were no means of prolonging valuable fruits. Claiming that his ideas were now “beyond the reach of controversy,” Knight declared that fruits “under our ordinary modes of propagation by grafts and buds, all become subject within no very distant period to the debilities and diseases of old age” (ibid., p. 323). Knight’s certainty even extended beyond the grave. The author of Knight’s memorial sketch written after his death in 1838 assured his readers that Knight’s ideas about the dangers of propagating old varieties were “now almost universally adopted” (ibid., p. 12). This was hardly the case—Philips’s trip to Covent Garden became a recurrent point of debate in the agricultural press, with contributors taking both sides of the controversy. Timothy Pickering, the American politician who founded an agricultural society in the Boston area after his retirement from politics, maintained against Philips that “Mr Knight’s doctrine was founded on *facts*, and could not therefore be overthrown. The individual instances mentioned in opposition are only exceptions to the general principle” (1826, p. 339). Did a few healthy apples count against Knight’s doctrine? Did many healthy apples disprove it?

The fear that Knight’s ideas were contributing to the neglect and decay of varieties stuck—particularly in America, where Knight’s ideas had been debated and applied just as they were in Britain—contributing to a demand for new varieties in American nurseries, as commentators then observed (Towers 1853, p. 194; Pauly 2007, pp. 65–66). The American landscape designer Andrew Jackson Downing complained of the influence Knight had had in his country, writing: “We have not the slightest doubt that the fine kinds of fruit have only become ‘miserable outcasts’ from having been carelessly and improperly propagated.” Downing proposed instead that the ideas of Erasmus Darwin were sound, that trees were best understood as a “swarm” of individuals, and that buds are “entirely and decidedly distinct individual plants” (1837, p. 91).

For Downing, sufficient time had elapsed by 1837 to demonstrate the falsehood of Knight’s ideas. And this idea that the passage of time was reason enough to discount his theory grew increasingly pervasive. The Scottish botanist Robert Hogg echoed the growing opinion that the predicted decline had not come, and that the theory was entirely false:

It is now considerably upwards of half a century since this doctrine was first promulgated, and though the old, exhausted and diseased trees of the Herefordshire orchards, of which Mr Knight sooke, together with their *diseased* progeny—now that they have performed their part, and fulfilled the end of their existence—may ere this have passed away, we have the Golden Pippin still, in all the luxuriance of early youth. (1851, p. 97)

The shifting opinion was reflected also in changes to the *Encyclopaedia Britannica.* In the seventh edition, Knight’s ideas were still put forward as orthodox in the entry on “horticulture”: “It is well known that some of the favourite cider apples of the seventeenth century have become extinct, and others are fast verging into decrepitude” (Neil 1838, p. 24). In the 1856 edition of the *Encyclopaedia Britannica,* the entry for “horticulture” was updated to explain that “no deterioration has taken place in even our oldest fruits” (Neil and Macintosh, 1856, p. 702).

## Conclusion

Knight advanced a way of thinking about plant heredity that was, to his peers, entirely new and held immediate practical consequences for agricultural practice. For several decades, he was successful in convincing the public (and also himself) that Britain’s fruit trees were facing imminent decline. By the time of his death in 1837, a more consistent climate had alleviated these fears. This article has shown that good weather and high yields led to a gradual rejection of this idea. However, Knight’s doctrine survived for many decades particularly because both the agricultural press and the state lacked the means to produce data that could be used to decide the question as to the health of Britain’s fruit trees, or even to assess whether or not there were more “old” varieties being cultivated than new. Debates over the nature of grafts would continue for decades in the agricultural press, and the questions that Knight had raised about grafts remained unanswered: over time, business clearly went back to normal. The early decades of the nineteenth century are a crucial period in debates over the laws of heredity: historians have increasingly appreciated the role played by the wider public in providing the forms of evidence that informed these debates, but there has been little attention up until now as to the impacts of climate upon people’s thought about heredity.

As John Waller has argued, hereditary explanations of illness gained popularity with doctors in the nineteenth century in part because of the inability to gain ground on treating conditions viewed as incurable (2002, pp. 445–446). Hereditary explanations, Waller shows, appealed to older ideas about the constitution of the individual, and thereby helped to preserve the reputation of medical practice (p. 414). In this paper, I have argued that hereditary explanations were not the only way of managing incurable diseases during this period: old age itself, and the decline of old varieties, also provided a means of delineating what the art of gardeners (and doctors) can and cannot achieve. Canker was both poorly understood and resistant to treatment. While Knight rejected the idea that hereditary ailments plagued trees propagated by grafts, he remained insistent that the appearance of the disease had little to do with the poor weather that appeared to be the obvious culprit for the decline in Britain’s fruit trees. New varieties were cultivated and marketed as being intrinsically healthy. What was promised, in effect, was freedom from the vagaries of climate, and the development of stock that could transmit youth itself, instead of the debilities of old age.

Knight provided an elegant explanation of canker, and his description of grafted trees as continuations of the same individual was easily comprehended by the wider agricultural readership. Knight was accused of being a hobbyist and lacking practical experience, but he was never charged with putting forward esoteric or obscure ideas. One might expect that his ideas would remain popular until competing understandings of grafts gained in popularity; however, I have argued instead that Knight’s ideas succumbed to good weather. Improved climate and better harvests seem to have been the main evidence against Knight’s ideas. While the historiography of heredity includes copious discussion of the *idea* of climate as a causal factor in variation and modification, far fewer works have attempted to assess what effect, if any, changes in climate have had on popular understandings of heredity. In the case of the golden pippin, poor weather and changes in climate provided Knight with an opportunity to put forward what were new and radical ideas about the nature of plant life. He challenged popular understandings of plant heredity and, drawing upon Malthusian concerns of providing for an increasing population, he used his institutional powers to shift the practices of farmers, plant breeders, and nurseries (at least for a time).

## Footnotes

1 This is not to say that gardeners, seed companies, and nurserymen were without agency and influence during this period. Easterby-Smith (2015, p. 184) has looked at the technical knowledge wielded by nurserymen in the eighteenth century in shipping plants and seeds, demonstrating that this expertise granted them strong influence on the science of botany and on wealthy amateurs. Histories of seed companies have drawn links from experiments in hybridization and plant breeding carried out by commercial companies to the thoughts of Darwin, Galton, and other authorities (Gayon and Zallen 1998, p. 248).

2 See ‘Letters from Thomas Andrew Knight to Sir Joseph Banks, 1796–1805’, held at the Natural History Museum Archives, London. Hereafter MSS/KNI.

3 Fulford et al. (2011–).
